# Three decades of coronary intravascular ultrasound and optical coherence tomography: A structured narrative review of published bibliometric evidence and trial-driven research phases

**DOI:** 10.1016/j.ahjo.2026.100991

**Published:** 2026-10-24

**Authors:** Syed Nazar Raza

**Affiliations:** Department of Allied and Healthcare Sciences, SAHSR, Jamia Hamdard, New Delhi, 110025, India

**Keywords:** Intravascular ultrasound, Optical coherence tomography, Intracoronary imaging, Percutaneous coronary intervention, Narrative review, Research evidence mapping

## Abstract

**Background:**

Intravascular ultrasound (IVUS) and optical coherence tomography (OCT) now sit at the centre of invasive coronary assessment, and intracoronary imaging has been elevated to a Class 1 recommendation for guiding percutaneous coronary intervention (PCI) in complex lesions. Several bibliometric analyses have mapped adjacent literatures, but their findings have never been assembled and appraised together for the coronary IVUS and OCT field.

**Objectives:**

To assemble, appraise, and synthesise the published bibliometric evidence describing the coronary IVUS and OCT literature; to place that evidence alongside the trials, technologies, and guideline events that plausibly drove its inflection points; and to specify a reproducible protocol for the formal bibliometric analysis this field still lacks.

**Methods:**

Structured narrative review reported against the SANRA quality criteria, with search reporting adapted from PRISMA-ScR. PubMed/MEDLINE, Scopus, and Web of Science Core Collection were searched from 1 January 1990 to the date of the final search, using two purpose-built search strands: one for published bibliometric or scientometric analyses of intracoronary and closely related optical or ultrasound imaging literatures, and one for the primary trials, meta-analyses, registry studies, and guidelines used to date and interpret literature inflection points. No article-level records were exported to bibliometric software, and no publication counts, citation counts, author or institution rankings, or network maps were generated by the present author. Every quantitative statement in this review is attributed to the source that reported it, ensuring transparency and traceability of the evidence.

**Findings:**

Thirty-eight sources were included, of which five were bibliometric analyses covering coronary or ophthalmic OCT, drug-coated balloons, and adjacent domains. None took coronary IVUS as its primary subject, which is itself the clearest finding of this appraisal: the quantitative mapping of this field is markedly OCT-weighted, and the IVUS literature has never been formally mapped. Within the OCT-focused analyses, Japan, the United States, and China were consistently the leading contributors, and the International Journal of Cardiology was the single most prolific outlet for OCT research in acute coronary syndromes. Read against the clinical record, the evidence supports a four-phase account of the field: an IVUS-dominant foundational phase, a comparative-growth phase following the clinical arrival of OCT, an outcomes-driven consolidation phase built on OPINION, ILUMIEN III, ULTIMATE, and IVUS-XPL, and a diversification phase from 2022 onward shaped by ILUMIEN IV, OCTOBER, OCTIVUS, RENOVATE-COMPLEX-PCI, IVUS-ACS, and OCCUPI, and closed by the 2025 guideline upgrade.

**Conclusions:**

The coronary IVUS and OCT literatures have followed distinct but converging trajectories punctuated by identifiable trial and technology events. The quantitative evidence describing those trajectories remains partial and modality-skewed. The prespecified protocol given here is offered so that the analysis this field needs can be executed and audited by others.

## Highlights

• Assembles and appraises, for the first time, the published bibliometric evidence bearing on the coronary IVUS and OCT literature.
• Documents a concrete gap: no bibliometric analysis to date takes coronary IVUS as its primary subject.
• Proposes a four-phase account of the field, anchored to named trials, technologies, and the 2025 Class 1 guideline upgrade.
• Reports the full search strategy and maps every principal claim to the source that supports it.
• Supplies a prespecified, reusable protocol for the formal bibliometric analysis the field still lacks.

## 1 Introduction

Coronary angiography renders a three-dimensional, diseased vessel as a two-dimensional shadow of its lumen. That compression is diagnostically costly: plaque composition, the true extent of vessel remodelling, and the mechanisms behind stent failure are all largely invisible to it [1], [2]. IVUS, which entered clinical practice around the turn of the 1990s, answered part of that problem by producing tomographic cross-sections of the vessel wall from a catheter-mounted transducer, making atheroma burden, remodelling, and stent apposition directly visible [1], [2]. Roughly a decade later OCT arrived as its optical counterpart, trading depth of penetration for an order-of-magnitude gain in axial resolution and thereby resolving fibrous-cap thickness, strut-level stent coverage, macrophage accumulation, and calcified nodules that ultrasound cannot reliably separate [3], [4]. Catheters that place both probes on a single shaft, or that pair ultrasound with near-infrared spectroscopy, now aim to recover the structural and compositional information of both modalities within one pullback [5], [6].

The clinical standing of the two techniques was settled by a long sequence of randomised comparisons. OPINION and ILUMIEN III established OCT as a workable alternative to IVUS for procedural guidance [7], [8], [9]; ULTIMATE, IVUS-XPL, and the Pan-London cohort demonstrated the advantage of imaging-guided over angiography-guided stenting [10], [11], [12], [13]; and the 2023 to 2024 wave of trials, comprising RENOVATE-COMPLEX-PCI, ILUMIEN IV, OCTOBER, OCTIVUS, IVUS-ACS, and OCCUPI, extended that evidence into complex lesions, bifurcations, and acute coronary syndromes [14], [15], [16], [17], [18], [19]. Two network meta-analyses published in 2024 synthesised the randomised evidence available up to 2023; neither could include IVUS-ACS or OCCUPI, and the literature search for one of them closed on 30 August 2023 [20]. Their endpoints, and therefore their conclusions, were not the same. Stone and colleagues took target-lesion failure as the primary endpoint and found it lower with imaging guidance than with angiography guidance [20]. Giacoppo and colleagues instead used target-lesion revascularisation and myocardial infarction as coprimary endpoints; target-lesion revascularisation was significantly reduced with IVUS compared with angiography but not with OCT compared with angiography, and myocardial infarction did not differ significantly between any of the guidance strategies [21]. In February 2025 the ACC/AHA/ACEP/NAEMSP/SCAI acute coronary syndrome guideline raised intracoronary imaging in complex lesions from a Class 2a to a Class 1 (Level of Evidence A) recommendation, the strongest institutional endorsement the field has received [22].

A scientific literature accumulated alongside that clinical record, and bibliometric methods offer one way to describe how. Quantitative mapping of publication output, geography, collaboration, and thematic structure has been applied productively to retinal and ophthalmic OCT [23], [24], to OCT in acute coronary syndromes [25], to OCT across cardiovascular disease [26], and to neighbouring interventional technologies such as drug-coated balloons [27]. What has not been done is to bring those separate mappings together and ask what they collectively establish, and fail to establish, about coronary IVUS and OCT.

That is the task of the present review, and it is deliberately a narrower and more verifiable one than a primary bibliometric analysis. This article does not generate publication counts, citation counts, productivity rankings, or co-occurrence networks. It does four things instead. First, it identifies and appraises the bibliometric analyses that bear on this field, and reports what each of them actually measured (Section 3, Table 1). Second, it sets out an explicit, criterion-based account of the trials and technologies that mark the phases of the field, extended through 2025 (Section 4, Table 2, Fig. 1). Third, it examines the persistent distance between the outcomes evidence and real-world utilisation (Section 7). Fourth, it specifies a protocol, in enough detail to be executed by another investigator, for the formal bibliometric analysis this field still lacks (Section 9, Table 4).

**Fig. 1:**
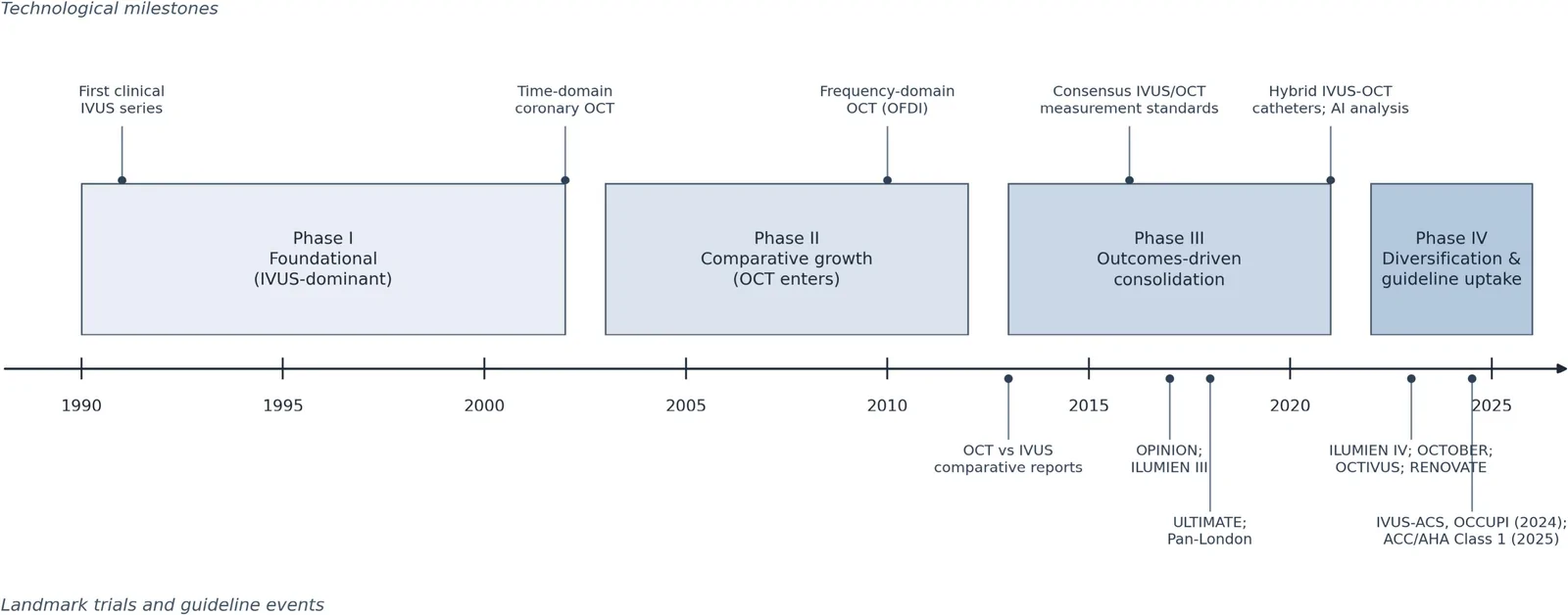
Schematic timeline of the four phases described in Section 4, with technological milestones above the axis and landmark trials and guideline events below it. The figure contains no measured quantity: no publication counts, citation counts, or growth curves are plotted, and the phase bands mark interpretive boundaries only.

## 2 Methods

### 2.1 Design and reporting standards

This is a structured narrative review. It was prepared against the SANRA criteria for narrative review quality [28], and its search and selection reporting follows the items of the PRISMA extension for scoping reviews that apply to a single-author narrative synthesis [29]; a full PRISMA 2020 flow was not appropriate because the review does not pool effect estimates [30]. No protocol was registered in advance, which is stated here rather than left to be inferred.

### 2.2 Information sources and search strategy

PubMed/MEDLINE, Scopus, and the Web of Science Core Collection were searched from 1 January 1990 onward. All three databases were last searched on 15 August 2026, and no source published after that date is cited. Two search strands were run separately, because the two categories of evidence serve different purposes in the synthesis.

#### 2.2.1 Strand A (bibliometric evidence)

The PubMed formulation was: (“intravascular ultrasound”[tiab] OR IVUS[tiab] OR “optical coherence tomography”[tiab] OR “optical frequency domain imaging”[tiab] OR “intracoronary imaging”[tiab] OR “intravascular imaging”[tiab]) AND (coronary[tiab] OR “percutaneous coronary intervention”[tiab] OR PCI[tiab] OR stent*[tiab] OR atherosclero*[tiab]) AND (bibliometric*[tiab] OR scientometric*[tiab] OR “research trends”[tiab] OR “publication trends”[tiab] OR hotspot*[tiab] OR “co-citation”[tiab] OR “knowledge map*”[tiab] OR VOSviewer[tiab] OR CiteSpace[tiab] OR bibliometrix[tiab]). The Web of Science formulation replaced [tiab] with TS=, and the Scopus formulation used TITLE-ABS-KEY, with the same Boolean structure and no other changes. Bibliometric analyses from adjacent domains, which serve as comparators, would not be captured by the coronary block, so two supplementary queries were run in the same databases. The first retained the imaging and bibliometric blocks but dropped the coronary block, which brought in ophthalmic and retinal OCT analyses; the second paired the bibliometric block with (“drug-coated balloon”[tiab] OR “drug-eluting balloon”[tiab]). Hits from both queries were kept only if they met the comparator criterion given in Section 2.3, and the reference lists of the two coronary analyses [25], [26] were also checked for further candidates.

#### 2.2.2 Strand B (clinical and technical anchors)

The same imaging and coronary blocks were combined with a third block of (“randomized controlled trial”[pt] OR “meta-analysis”[pt] OR guideline[pt] OR “practice guideline”[pt] OR registry[tiab] OR “national inpatient sample”[tiab] OR Medicare[tiab] OR “consensus document”[tiab]). Reference lists of the retrieved trials, network meta-analyses, and guidelines were hand-searched for pivotal reports that the electronic search had missed; the primary ILUMIEN III report [8] was identified this way.

Both strands were restricted to English-language publications. The complete search outputs, including the number of records retrieved per database and per strand, are available from the author on request. The PubMed Strand A search returned 9 records in the current search run. A combined record count across both strands and the comparator queries was not calculated in this update; therefore, duplicate-removal and full-text assessment counts are not reported here. Thirty-eight sources were included in the final narrative synthesis.

### 2.3 Eligibility criteria

For Strand A, a source was eligible if it reported an original quantitative analysis of a body of literature, using an article-level dataset exported from a bibliographic database, in a domain that was either coronary intracoronary imaging itself or a closely adjacent optical or catheter-based imaging field usable as a comparator. Reviews that described bibliometric findings without generating them were excluded, as were analyses of unrelated clinical domains.

For Strand B, eligible sources were randomised trials of imaging-guided PCI, systematic reviews and network meta-analyses of those trials, national registry or administrative-database analyses of imaging utilisation, society guidelines, and consensus or technical reports establishing measurement standards or describing device generations. Single-patient case reports, conference abstracts without full publication, editorials, and commentaries were not cited.

### 2.4 Selection, charting, and claim-to-source mapping

Records were screened by title and abstract and then in full text by the single author, who also performed data charting. For each Strand A source the following fields were extracted: subject domain, coverage period, database used, analytical software, number of publications analysed, and the leading countries, institutions, and journals as reported by that source. These fields populate Table 1. For Strand B, the extracted fields were trial or study name, design, comparison, primary result, and year.

Because this review makes descriptive claims about a literature it did not itself measure, each principal claim is tied explicitly to the source that supports it. Table 3 sets out that mapping, distinguishing statements that reproduce a reported quantity, statements that generalise across several sources, and statements that are the present author's interpretation and carry no external quantitative support. Single-author screening and charting is a limitation and is treated as such in Section 10.

### 2.5 Post-search additions

The search window was fixed so that every source cited here, including the 2025 guideline [22] and the 2025 bibliometric analysis of drug-coated balloon research [27], lies within the dated search described in Section 2.2. No source was added after the search was completed.

### 2.6 What this review does and does not claim

No article-level dataset was exported from any database by the present author. VOSviewer, CiteSpace, and bibliometrix were not run. No publication count, citation count, h-index, country ranking, institutional ranking, journal ranking, co-authorship network, co-citation network, or keyword co-occurrence map presented anywhere in this manuscript is an original product of this work; each is either reproduced with attribution from a cited bibliometric analysis or is absent. Fig. 1 is a schematic of phase boundaries and dated events and contains no measured quantity of any kind. Readers should treat the whole of 3, 4, 5, 6, 7 as a synthesis of previously published findings.

## 3 The published bibliometric evidence base

Five sources met the Strand A criteria. Their scope, methods, and headline findings are set out in Table 1. Two address coronary applications of OCT directly [25], [26]; two address ophthalmic OCT and are included only as comparators for the growth behaviour of an optical imaging technology [23], [24]; one addresses drug-coated balloons and is included as a comparator for an interventional-cardiology device literature with a similar adoption profile [27].

**Table 1:** Published bibliometric analyses meeting the Strand A eligibility criteria. All values are as reported by the cited source; none was recalculated, verified against the underlying records, or generated by the present author.

Source	Domain and coverage	Database and tools	Publications analysed	Reported leading contributors and outlets
Zhang et al., 2024 [25]	OCT in acute coronary syndromes; 1998–2023	Web of Science Core Collection; CiteSpace, VOSviewer	965	Japan 20.8% (*n* = 201), United States 19.9% (*n* = 192), China 14.1% (*n* = 136); highest citation total in the United States; Harvard Medical School and Harbin Medical University lead institutions; International Journal of Cardiology the most prolific journal; plaque erosion, calcified nodules, and intracoronary imaging identified as current frontiers
Lian et al., 2024 [26]	OCT across cardiovascular disease, paired with a meta-analysis of OCT- versus angiography-guided PCI; 2003–2023	Web of Science Core Collection; VOSviewer, CiteSpace, R	310	Eight keyword clusters including coronary artery disease, deep learning, coronary stenosis, and plaque rupture; most productive authors predominantly United States based, with one leading Chinese author
Lin et al., 2022 [23]	Retinal OCT; 1991–2021	Web of Science; keyword co-occurrence and co-citation analysis	25,175	Five hotspot clusters; emerging frontiers in OCT angiography, vessel density, and deep learning
Qian and Wang, 2024 [24]	Ophthalmic OCT and OCT angiography; 2003–2022	Web of Science Core Collection; standard bibliometric tools	20,817 (48,160 authors, 106 countries)	United States most productive and most cited; Investigative Ophthalmology and Visual Science the leading journal; Moorfields Eye Hospital the leading institution
Yu et al., 2025 [27]	Drug-coated balloons in coronary artery disease; 2004 to mid-2025	Web of Science, Scopus, PubMed; CiteSpace, VOSviewer, bibliometrix	1092 (from 2314 retrieved)	Output from 55 countries and 871 institutions; sustained annual growth from 2011; steep recent expansion of Chinese output

### 3.1 What this evidence base does not cover

The single most consequential observation from Table 1 is an absence. Every coronary bibliometric analysis identified treats OCT as its subject [25], [26]. None takes IVUS as its primary subject, despite IVUS being the older modality, the one with the larger accumulated randomised evidence base, and the one that accounts for the substantial majority of intracoronary imaging performed in practice [31], [32]. The quantitative description of this field is therefore skewed towards its newer technology, and the claim that IVUS output has matured or plateaued, which recurs in narrative accounts including this one, currently rests on no published measurement at all.

Two further limits of the evidence base deserve statement. All five analyses draw principally on the Web of Science Core Collection, so their coverage inherits the indexing boundaries of that database; the drug-coated balloon analysis is the only one to triangulate across three databases [27]. And all five attribute publications by author affiliation, which credits multinational trials to whichever countries supplied the listed authors rather than to the sites that recruited patients. For a field whose signature studies are international, that is a material distortion, and it is a reason to treat country-level statements as approximate.

## 4 A four-phase account of the field

### 4.1 Criteria for designating a landmark

The criteria for designating a landmark were set before the trial literature was charted. A publication qualified if it belonged to any one of four categories, each of which was sufficient on its own: a multicentre randomised trial with a clinical primary endpoint comparing imaging-guided with angiography-guided PCI, or one imaging modality with the other; a systematic review or network meta-analysis pooling such trials; a society guideline in which the recommendation class for intracoronary imaging changed; or a consensus document whose measurement standards were later adopted as reporting conventions. Two further kinds of entry appear in Table 2 without being designated landmarks under this rule. For Phases I and II, which predate randomised comparisons, the foundational descriptive work [1], [2] and the first OCT-versus-IVUS reports [33] are listed as the events that defined each phase. The Pan-London cohort [13] is listed because it extends the outcome comparison of imaging-guided and angiography-guided PCI to a large unselected population treated in routine practice. Technological milestones are recorded in a separate column and were not subject to the rule. On this basis the framework includes ILUMIEN IV, OCTOBER, OCTIVUS, RENOVATE-COMPLEX-PCI, IVUS-ACS, and OCCUPI, and ILUMIEN III is cited through its primary randomised report [8] together with its one-year follow-up [9]. Table 2 sets out the resulting framework and Fig. 1 shows it along a time axis.

**Table 2:** Four-phase framework with landmark designations applied according to the criteria in Section 4.1. Phase boundaries are interpretive; the dated events within each phase are not.

Phase	Technological milestone	Landmark trials and guideline events	Character of the literature
I. Foundational 1990–2002	Clinical introduction of coronary IVUS	Consensus IVUS measurement standards; early atherosclerosis-regression and stent-optimisation studies [1], [2]	Descriptive and single-modality; no comparator available
II. Comparative growth 2003–2012	Time-domain coronary OCT	OCT methodology and terminology consensus, 2010 [4]; first OCT-versus-IVUS comparative reports [33]	A second literature forms beside a continuing IVUS output
III. Outcomes-driven consolidation 2013–2021	Frequency-domain OCT and optical frequency domain imaging	OPINION [7]; ILUMIEN III primary report [8] and one-year follow-up [9]; ULTIMATE [10] and its three-year outcomes [11]; IVUS-XPL five-year follow-up [12]; Pan-London PCI cohort [13]	Citation-dense; organised around the modality-versus-modality question
IV. Diversification and guideline uptake 2022 onward	Hybrid IVUS-OCT catheters [5], [6]; artificial-intelligence-assisted image analysis	RENOVATE-COMPLEX-PCI [14]; ILUMIEN IV [15]; OCTOBER [16]; OCTIVUS [17]; IVUS-ACS [18]; OCCUPI [19]; network meta-analyses [20], [21]; 2024 ESC chronic coronary syndrome guideline [34]; 2025 ACC/AHA Class 1 upgrade [22]	Question shifts from which modality to which patients, which lesions, and which systems

**Table 3:** Mapping of each principal claim in this review to its supporting source, with the status of the claim stated explicitly. Claims marked as author interpretation are hypotheses to be tested by the analysis specified in Section 9, not findings of this review.

Principal claim in this review	Status of the claim	Supporting source(s)
Japan, the United States, and China lead output in coronary OCT research, with the reported shares of 20.8%, 19.9%, and 14.1% of 965 publications	Reproduced quantity	Zhang et al. 2024 [25]
The International Journal of Cardiology is the most prolific journal for OCT research in acute coronary syndromes	Reproduced quantity	Zhang et al. 2024 [25]
Harvard Medical School and Harbin Medical University lead institutional output and citation in the OCT / acute coronary syndrome literature	Reproduced quantity	Zhang et al. 2024 [25]
Cardiovascular OCT output grew from a low base after 2003 across 310 analysed publications; thematic clusters include deep learning and plaque rupture	Reproduced quantity	Lian et al. 2024 [26]
The United States is the most productive and most cited country in ophthalmic OCT, across 20,817 articles from 106 countries	Reproduced quantity (adjacent domain, used as comparator only)	Qian and Wang 2024 [24]; Lin et al. 2022 [23]
Germany, China, Italy, Japan, and the United States dominate drug-coated balloon research, with steep recent growth in Chinese output	Reproduced quantity (adjacent domain, used as comparator only)	Yu et al. 2025 [27]
No published bibliometric analysis takes coronary IVUS as its primary subject	Negative finding of the present search, valid to the search date	Absence across [23], [24], [25], [26], [27]; Section 2.2
IVUS-guided PCI rose from 3.4% to 8.7% of PCIs for myocardial infarction between 2008 and 2019, and OCT from 0.0% to 0.6%	Reproduced quantity	Park et al. 2023 [31]
Intravascular imaging use in Medicare PCI rose from 9.5% in 2013 to 15.4% in 2019	Reproduced quantity	Fazel et al. 2023 [32]
The four-phase account of the field and its boundary years	Author interpretation; no external quantitative support	Section 4; Fig. 1; Table 2
IVUS-specific publication output has plateaued while OCT output has grown more steeply	Author interpretation, consistent with but not measured by the cited sources	4.6, 8; see Limitations

### 4.2 Phase I, foundational, approximately 1990 to 2002

IVUS held the field alone. Output was dominated by the description of what tomographic imaging revealed that angiography could not: atheroma burden, compensatory remodelling, and the mechanical determinants of stent expansion [1], [2]. The research question of the period was largely descriptive, and the modality had no competitor against which to be compared.

### 4.3 Phase II, comparative growth, approximately 2003 to 2012

Time-domain OCT entered coronary practice and the first head-to-head comparisons with IVUS appeared [33]. The expert consensus document on OCT methodology and terminology published in 2010 [4] gave the newer modality the measurement conventions that made comparative work interpretable. IVUS output continued at a steady volume while a second, smaller literature formed beside it.

### 4.4 Phase III, outcomes-driven consolidation, approximately 2013 to 2021

Frequency-domain acquisition removed the practical obstacles that had constrained time-domain OCT, and the field turned from description to outcomes. OPINION established non-inferiority of optical frequency domain imaging to IVUS for procedural guidance [7], ILUMIEN III compared OCT, IVUS, and angiography for stent implantation [8], [9], and ULTIMATE, IVUS-XPL, and the Pan-London cohort established the advantage of imaging guidance over angiography alone at progressively longer follow-up [10], [11], [12], [13]. This is the citation-dense core of the field.

### 4.5 Phase IV, diversification and guideline uptake, 2022 onward

The 2023 to 2024 trials asked narrower and harder questions than their predecessors. RENOVATE-COMPLEX-PCI showed benefit from imaging guidance in complex lesions [14]. ILUMIEN IV produced a larger minimum stent area with OCT guidance but did not reduce two-year target-vessel failure [15], a split result that subsequently accounted for much of the inconsistency detected in network meta-analysis [21]. OCTOBER found benefit in complex bifurcations [16], OCTIVUS found OCT non-inferior to IVUS across a diverse lesion population [17], IVUS-ACS extended the case to acute coronary syndromes with a substantial reduction in one-year target-vessel failure [18], and OCCUPI confirmed superiority of OCT guidance over angiography in complex lesions [19]. Two network meta-analyses consolidated the field in 2024 [20], [21], and the 2025 guideline upgrade to Class 1 [22] closed the phase.

The distinguishing feature of this phase is that the modality-versus-modality question, which organised Phase III, has largely been answered and has given way to questions about which patients, which lesions, and which health systems. That shift is visible in the trial designs themselves, and it is the reason the phase is labelled diversification rather than growth.

### 4.6 A caution about the phase model

The phases above are an interpretive device, not a measurement. Their boundaries were placed by judgement, informed by the dating of the events in Table 2 and by the growth behaviour reported for adjacent optical imaging literatures [23], [24], and different defensible boundaries could be drawn. Whether the underlying publication series actually shows inflections at these points is precisely the question that the protocol in Section 9 is designed to answer, and it is possible that a formal analysis will not support them.

## 5 Geographic, journal-level, and institutional patterns

What can be said here is limited to what the sources in Table 1 measured, and is reported accordingly. For coronary OCT in acute coronary syndromes, Zhang and colleagues analysed 965 publications from 58 countries and 1389 institutions indexed in the Web of Science between 1998 and 2023, and reported Japan as the leading contributor at 20.8% of output, followed by the United States at 19.9% and China at 14.1%, with the United States accumulating the highest citation total [25]. Harvard Medical School and Harbin Medical University led both publication count and citation count among institutions, and the International Journal of Cardiology was the single most prolific journal [25]. The broader cardiovascular OCT analysis of Lian and colleagues, covering 310 publications between 2003 and 2023, likewise found the most productive authors to be predominantly United States based, with one leading Chinese author [26].

No comparable measurement exists for coronary IVUS, and none is offered here.

The concentration pattern that the OCT analyses do show is not unusual for an imaging technology. In ophthalmic OCT, across 20,817 articles from 106 countries, the United States was both the most productive and the most cited country and a single institution led output [24]; across 25,175 retinal OCT publications the same concentration is visible [23]. In drug-coated balloon research, Germany, China, Italy, Japan, and the United States dominate, with Chinese output expanding steeply in the most recent years [27]. The plausible explanations, differential device-approval timelines, reimbursement environments, and national investment in interventional research capacity, are consistent across these fields but have not been tested in any of them.

## 6 Thematic research fronts

The thematic structure described below is drawn from the keyword and cluster analyses reported in the cited bibliometric sources, supplemented by the subject matter of the trials in Table 2. It was not derived by co-word analysis in the present work.

### 6.1 Plaque characterisation and vulnerable-plaque biology

The oldest front and still an active one, running from IVUS-based atheroma quantification and virtual-histology tissue characterisation to OCT identification of thin-cap fibroatheroma, plaque erosion, and calcified nodules [1], [2], [3], [4]. Zhang and colleagues identified plaque erosion and calcified nodules as among the most recent frontiers in the OCT literature [25], and plaque rupture emerged as a distinct cluster in the analysis of Lian and colleagues [26].

### 6.2 Procedural guidance and stent optimisation

Minimum stent area, malapposition, edge dissection, and sizing algorithms. This front expanded with drug-eluting stent adoption and supplies the mechanistic rationale for the outcome trials [10], [12], [13]. ILUMIEN IV is instructive here, because it separated the procedural result, a larger minimum stent area, from the clinical one, which did not follow [15].

### 6.3 Comparative outcomes research

The trial programmes of Table 2 and the meta-analyses that pool them [20], [21]. This is the citation-dense centre of the field and the direct cause of the 2025 guideline change [22].

### 6.4 Computational analysis and hybrid acquisition

The newest front, covering automated plaque segmentation, deep-learning lesion classification, and dual-probe IVUS-OCT catheters [5], [6]. Deep learning appeared as a distinct keyword cluster in the cardiovascular OCT analysis [26], and emerged as a frontier term in retinal OCT some years earlier [23], which suggests the coronary field is following a path the ophthalmic field has already taken.

## 7 The distance between evidence and practice

A literature and its clinical uptake are separable things, and in this field the separation is wide enough to be worth quantifying. Analysing 2,881,746 PCIs for myocardial infarction in the United States National Inpatient Sample, Park and colleagues found IVUS guidance rising from 3.4% of procedures in 2008 to 8.7% in 2019, and OCT-guided procedures increasing by 548% between 2011 and 2019 while still reaching only 0.6% of all such PCIs; imaging-guided procedures carried lower adjusted odds of in-hospital mortality, at 0.66 (95% confidence interval 0.60 to 0.72) [31]. In a Medicare cohort of 1,189,470 patients, Fazel and colleagues found intravascular imaging used in 10.5% of PCIs overall, rising from 9.5% in 2013 to 15.4% in 2019, with substantial regional variation and an association with lower major adverse cardiovascular events [32].

So at the close of the period in which the outcomes evidence became decisive, roughly six in seven procedures in these cohorts were still performed without imaging. The 2025 Class 1 recommendation [22] gives the field a natural experiment: if guideline strength drives utilisation, the effect should be detectable in registry data for 2025 onward, and if it drives publication, it should be detectable as a burst in implementation and cost-effectiveness keywords. Both are testable, and neither has been tested. The implementation and health-economics literature specific to intracoronary imaging appears in none of the analyses in Table 1, which makes it the most obvious candidate for the first formal bibliometric treatment of this field.

## 8 Discussion

Three points seem worth drawing out. The first concerns what the appraisal in Section 3 actually establishes. It is easy to assume that a field with three decades of history and a Class 1 guideline recommendation has been quantitatively described, and the assumption is wrong. The bibliometric coverage of intracoronary imaging is thin, recent, OCT-weighted, and reliant on a single indexing database. Statements about the shape of this literature, have been circulating without measurement behind them. Identifying that gap precisely is a more useful contribution than restating the assumptions would have been.

The second concerns the relationship between trials and literature. The phase model of Section 4 assumes that pivotal trials drive publication activity, and the assumption is plausible but untested, and there is at least one reason to doubt its simplicity. ILUMIEN IV returned a partly negative result [15], yet a negative result in a large trial typically generates more subsequent literature rather than less, through secondary analyses, editorials, and reconciliation attempts, one of which is visible in the network inconsistency reported by Giacoppo and colleagues [21]. A citation-burst analysis around 2023 would distinguish the two mechanisms, and would test whether trial outcome direction predicts literature response at all.

The third concerns the apparent maturity of IVUS. The intuitive reading of a flat IVUS publication series is that the modality has been superseded. A more likely reading is that IVUS methodology has become sufficiently standardised that its research contribution now appears inside comparative and outcomes studies rather than as IVUS-specific methodological papers, so that the modality is undercounted by any keyword-based approach that looks for it by name. IVUS-ACS enrolled 3505 patients and reported a 45% relative reduction in one-year target-vessel failure [18], which is not the profile of a superseded technology. Testing this properly requires comparing keyword-indexed IVUS output against full-text mentions of IVUS within the broader PCI literature, a comparison that Section 9 accordingly specifies.

These three points share a structure: each is a claim that this review can pose but cannot settle. That is the honest position of a narrative synthesis of a partially measured field, and it is the reason the protocol below is included as a deliverable rather than gestured at as future work.

## 9 A prespecified protocol for a formal bibliometric analysis

Table 4 sets out a protocol at sufficient specificity to be executed by an independent investigator and audited against this manuscript. It is designed to answer the three questions raised in Section 8: whether the phase boundaries of Section 4 correspond to inflections in the publication series, whether trial events produce detectable citation bursts, and whether IVUS output is undercounted by keyword-based retrieval.

**Table 4:** Prespecified protocol for the formal bibliometric analysis of the coronary IVUS and OCT literature, including three falsifiable hypotheses derived from the interpretations advanced in this review.

Element	Prespecified specification
Databases	Web of Science Core Collection and Scopus, exported separately and deduplicated by DOI and then by fuzzy title match. PubMed used as a coverage check on the deduplicated set, not as a primary source, because it lacks cited-reference data.
Search string	The Strand A imaging and coronary blocks of Section 2.2, without the bibliometric block, applied to title, abstract, and author keywords. Modality strands run separately as well as jointly so that IVUS-only, OCT-only, and joint output can be distinguished.
Coverage and record types	1 January 1990 to 31 December of the year preceding analysis. Articles and reviews retained; editorials, letters, meeting abstracts, and errata excluded and the excluded counts reported.
Software	bibliometrix in R for indicators and country and institution attribution [35]; VOSviewer for co-authorship, co-citation, and keyword co-occurrence maps [36]; CiteSpace for citation-burst and timeline analysis [37]. Versions and parameter settings reported.
Primary indicators	Annual and cumulative output by modality; compound annual growth rate by phase; country, institution, author, and journal productivity with citation impact; Bradford and Lotka distributions; international collaboration index.
Structural analyses	Keyword co-occurrence with thematic evolution across the four phases of Table 2; document co-citation clustering with silhouette and modularity reported; citation-burst detection with the burst-detection parameters stated in advance.
Prespecified hypothesis 1	Segmented regression of the annual output series will identify breakpoints within two years of the Phase II/III and Phase III/IV boundaries of Table 2. Failure to identify them falsifies the phase model.
Prespecified hypothesis 2	Citation bursts will be detected for the trial cohort of 2023 to 2024 [14], [15], [16], [17], [18], [19] irrespective of the direction of each trial result, indicating that trial salience rather than trial outcome drives literature response.
Prespecified hypothesis 3	The ratio of full-text IVUS mentions within the PCI literature to keyword-indexed IVUS records will rise across the four phases, consistent with the absorption hypothesis of Section 8 rather than with modality decline.
Sensitivity analyses	Repeat all indicators in each database separately to quantify the effect of database choice; repeat country attribution using corresponding-author country as well as all-author affiliation to quantify the multinational-trial distortion noted in Section 3.1.
Reporting and data availability	Reported against the bibliometric reporting guidance of Donthu and colleagues [38]. Search strings, export dates, record counts, deduplication logic, and the deduplicated record identifiers deposited in a public repository so that every figure can be reproduced.

## 10 Limitations

The governing limitation is stated in Section 2.6 and repeated here because it determines how everything else should be read: this review measures nothing. It reports what others have measured, appraises how they measured it, and adds an interpretive framework of its own. Any statement about publication volume, geography, journal concentration, or thematic structure carries the authority of its cited source and no more.

Beyond that, five limitations apply. Screening, selection, and charting were performed by a single author without duplicate assessment, so selection bias cannot be excluded and no inter-rater reliability can be reported. The search was restricted to English-language publications, which will under-represent Japanese, Chinese, and European-language contributions in a field where Japan is a leading contributor [25]. The bibliometric sources in Table 1 rely predominantly on the Web of Science Core Collection and inherit its indexing boundaries. Country attribution in those sources follows author affiliation and therefore misallocates the contribution of multinational trials. And the four-phase framework, the plateau interpretation for IVUS, and the trial-drives-literature assumption are the present author's inferences; Table 3 marks them as such, and Section 9 specifies how each could be falsified.

One further limitation is specific to the design chosen. A structured narrative review of bibliometric findings cannot resolve disagreements between its sources, because it lacks access to the underlying records. Where the sources in Table 1 differ in coverage period, database, or inclusion rule, as they do, this review can note the difference but cannot adjudicate it.

## 11 Conclusions

Coronary IVUS and OCT have followed distinct but converging trajectories, and the trial and guideline events that punctuate those trajectories can be dated precisely. The literature that accumulated alongside them has not been characterised with anything like the same precision. Five bibliometric analyses bear on this field, all of them recent, all OCT-weighted, and none taking coronary IVUS as its subject; the widely repeated claim that IVUS output has plateaued has never actually been measured.

This review therefore offers three things rather than a set of counts: an appraised map of what the bibliometric evidence does and does not establish, a criterion-based phase framework extended through the 2025 guideline upgrade, and a prespecified protocol with three falsifiable hypotheses. The value of the framework lies in its being testable, and the protocol exists so that someone, not necessarily this author, can test it.

## CRediT authorship contribution statement

**Syed Nazar Raza:** Writing – review & editing, Writing – original draft, Visualization, Validation, Supervision, Software, Resources, Project administration, Methodology, Investigation, Funding acquisition, Formal analysis, Data curation, Conceptualization.

## Clinical trial registration and reporting guidelines

Clinical trial registration is not applicable. The review was not registered on PROSPERO or any comparable register, which is stated in Section 2.1 of the manuscript. Reporting follows the SANRA criteria for narrative review quality, with search and selection reporting adapted from the applicable items of the PRISMA extension for scoping reviews.

## Ethics approval and consent

Ethical approval was not required. This article is a review of previously published literature. It involved no human participants, no animal subjects, no identifiable individual data, and no primary data collection of any kind. Consent to participate and consent for publication are therefore not applicable.

## Originality and prior publication

This manuscript is original work. It has not been published previously in whole or in part, is not under consideration by any other journal, and will not be submitted elsewhere while under consideration by this journal. No part of the manuscript has been posted to a preprint server. Its submission is approved by the sole author, and, where applicable, by the institution at which the work was carried out.

This is a revised version of a manuscript previously submitted to this journal under the title “Publication Trends in Intravascular Ultrasound and Optical Coherence Tomography for Coronary Imaging: A Bibliometric Analysis.” The title and study design have been revised in response to peer review, as set out in the accompanying point-by-point response to reviewers.

## Permissions and third-party material

The manuscript contains no material reproduced from any other source. Fig. 1 and Table 1, Table 2, Table 4 were created by the author for this article. Values reported in Table 1 are quantities reported by the cited studies and are attributed to them in the table, its legend, and the accompanying text; no figure, table, image, or text has been reproduced from any copyrighted work, and no permissions are therefore required.

## Funding

This research received no specific grant from any funding agency in the public, commercial, or not-for-profit sectors.

## Declaration of competing interest

The author declares that he has no known competing financial interests or personal relationships that could have appeared to influence the work reported in this paper.

The author has no affiliation with, consultancy for, speaker arrangement with, research support from, equity holding in, or patent interest relating to any manufacturer of intravascular ultrasound or optical coherence tomography systems, catheters, or related interventional devices.
